# Social, emotional and behavioural difficulties associated with persistent speech disorder in children: A prospective population study

**DOI:** 10.1002/jcv2.12126

**Published:** 2023-01-17

**Authors:** Yvonne Wren, Emma Pagnamenta, Faith Orchard, Tim J. Peters, Alan Emond, Kate Northstone, Laura Louise Miller, Susan Roulstone

**Affiliations:** ^1^ Bristol Speech and Language Therapy Research Unit North Bristol NHS Trust Bristol UK; ^2^ Bristol Dental School University of Bristol Bristol UK; ^3^ School of Psychology and Clinical Language Sciences University of Reading Reading UK; ^4^ School of Psychology University of Sussex East Sussex UK; ^5^ Centre for Academic Child Health Bristol Medical School Bristol UK; ^6^ Population Health Sciences Bristol Medical School Oakfield House University of Bristol Bristol UK; ^7^ Oakfield House University of Bristol Bristol UK; ^8^ Faculty of Health and Applied Sciences University of the West of England Bristol UK

**Keywords:** ALSPAC, antisocial behaviours, depression, emotional and behavioural difficulties, risk‐taking, social, speech disorder

## Abstract

**Purpose:**

Social, emotional and behavioural difficulties (SEBD) in childhood are associated with negative consequences across the life course. Children with developmental language disorder have been identified as being at risk of developing SEBD but it is unclear whether a similar risk exists for children with speech sound disorder, a condition which impacts on children's ability to make themselves understood and has been shown to be associated with poor educational outcomes.

**Methods:**

Participants were children who attended the 8‐year‐old clinic in the Avon Longitudinal Study of Parents and Children (*N* = 7390). Children with speech sound disorder that had persisted beyond the period of typical speech acquisition (persistent speech disorder [PSD]) at age 8 were identified from recordings and transcriptions of speech samples (*N* = 263). Parent‐, teacher‐ and child‐reported questionnaires and interviews including the Strengths and Difficulties Questionnaire, Short Moods and Feelings Questionnaire and measures for antisocial and risk‐taking behaviour were used to provide outcome scores for SEBD at 10–14 years in a series of regression analyses.

**Results:**

Following adjustment for biological sex, socio‐economic status and Intelligence Quotient, children with PSD at age 8 were more likely to show peer problems at age 10–11 years compared with their peers, as reported by teachers and parents. Teachers were more likely to report problems with emotionality. Children with PSD were no more likely to report symptoms of depression than their peers. No associations were observed between PSD, risk of antisocial behaviour, trying alcohol at age 10 or smoking cigarettes at age 14.

**Conclusions:**

Children with PSD may be at risk in terms of their peer relationships. This could impact on their wellbeing and, while not observed at this age, may lead to depressive symptoms in older childhood and adolescence. There is also the potential that these symptoms may impact on educational outcomes.

**Key points**

- Social, emotional and behavioural difficulties (SEBD) in childhood are associated with negative consequences in older childhood and adulthood.
- Children with developmental language disorder (DLD) have been identified as being at risk of developing SEBD.
- It is unclear whether a similar risk exists for children with Persistent Speech Disorder (PSD)—a condition which has been shown to impact on educational outcomes.
- Children with PSD are more likely to have problems with peer relationships.
- Children with PSD are more likely to show emotionality at school.
- While depressive symptoms are not more common in this population at age 8 years compared with peers, this may change in adolescence and poor peer relationships may contribute to this.
- Children with PSD are no more likely than their peers to show antisocial and risk‐taking behaviours.
- Future research should consider whether children with PSD are more or less likely to show SEBD as they get older and also which treatments work best to alleviate these if they are likely to present.
- Children with PSD or a history of PSD should be identified at school to ensure appropriate monitoring and support are in place.
- Speech and Language Therapists, education and health staff should be made aware that children with PSD are at risk of experiencing difficulties with peer relationships and emotionality in school and that the profile of these needs may change over time.
- Intervention for PSD in early childhood for speech may lead to a reduction in the negative sequelae seen in older childhood and adolescence.

## INTRODUCTION

There is a growing body of research on the individual and societal impact of childhood social, emotional and behavioural difficulties (SEBD), with evidence of immediate and long‐term consequences. This includes the development and continuity of internalising and externalising disorders (Agnew‐Blais et al., 2016; Kovacs & Devlin, 1998) and an increased risk of adverse outcomes across a range of domains of functioning. Emotional problems in childhood have been associated with increased risk of educational underachievement, unemployment, substance abuse, teenage pregnancy, poor physical health, and future suicidal behaviour (Bridge et al., 2006; Clayborne et al., 2019; Essau et al., 2014; Keenan‐Miller et al., 2007; Woodward et al., 2001). Similarly, behavioural problems in childhood are also predictive of a wide range of outcomes in adulthood, including future crime, substance use, mental health, risky sexual behaviour and violent partner relationships (Fergusson et al., 2005). The potential impact of SEBD on children's futures raises important questions about the aetiology of these difficulties, and how to identify children at risk.

‘Speech, language and communication needs’ (SLCN) is an umbrella term which encompasses difficulties with understanding and using language (such as vocabulary and grammar) as well as difficulties with the production and fluency of speech and the use of language in social contexts. SLCN are associated with a wide range of negative outcomes, including education, social participation and wellbeing and mental health (Botting et al., 2016; Durkin et al., 2017; Lee et al., 2020; McCormack et al., 2009; Snowling et al., 2006; Yew & O'Kearney, 2013). Specifically, an increased rate of conduct problems has been found in children with developmental language disorder (DLD) and adolescents with a history of DLD in comparison with typically developing peers (Conti‐Ramsden et al., 2013; St Clair et al., 2011) while other studies have highlighted the long‐term impacts of DLD on social, emotional and behavioural outcomes, friendships and bullying (Botting et al., 2016; Charman et al., 2015; Durkin et al., 2017; Lindsay & Dockrell, 2000; Norbury et al., 2016; van den Bedem, Dockrell, van Alphen, Kalicharan et al., 2018; Yew & O'Kearney, 2013). Associations have also been found between SLCN and mental health problems, such as anxiety and depression (Beitchman et al., 2001, 2014; Botting et al., 2016; Conti‐Ramsden & Botting, 2008; Wadman et al., 2011).

Theoretical frameworks for understanding the association between SLCN and SEBD are emerging. The quality of relationships may act as a risk or protective factor. For example, a qualitative study carried out with 11 children with speech and language disorders over the course of 6 months found that difficulties with relationships was a risk factor for wellbeing, whereas positive relationships appeared to be a protective factor (Lyons & Roulstone, 2018). Moreover, Forrest et al. (2018) found that emotional difficulties experienced by adolescents with DLD could be partially accounted for by peer problems at age 7. Other studies carried out with adolescents with DLD that have found an association with an increased risk of depression have suggested that a relationship between language difficulties and internalising symptoms may be moderated by bullying victimisation (Kilpatrick et al., 2019) or mediated by maladaptive emotional regulation strategies (van den Bedem, Dockrell, van Alphen, Kalicharan et al., 2018). A recent longitudinal study has also examined the developmental pathways that may mediate a relationship between DLD and externalising problems, suggesting a role for emotional competence (van den Bedem, Dockrell, van Alphen, de Rooij et al., 2018).

Whilst some types of SLCN such as DLD have been researched widely, much less is known about social, emotional and behavioural outcomes for children with speech sound disorder, a condition that impacts on children's production of speech and ability to make themselves understood. Furthermore, of the research that does exist, findings have been mixed and samples often include a wide range of speech difficulties. For example, McCormack et al. (2011) found increased rates of SEBD at ages 7–9‐years‐old while Beitchman et al. (2001) showed no difference compared to controls in adolescence. However, both studies used broad eligibility criteria and included children with a range of speech, voice and fluency difficulties rather than focussing exclusively on children with speech sound disorder. Lewis et al. (2016) looked specifically at children with speech sound disorder and found no association with symptoms of depression, anxiety, internalising or externalising problems in adolescence and adulthood. However, this study focussed on pre‐schoolers, an age at which speech sound difficulties in many children resolve within the period of typical speech acquisition. It is therefore possible that associations with SEBD may only be present for the subset of children who have speech sound disorder continuing beyond the period of typical speech acquisition (known as persistent speech disorder [PSD]). PSD has an estimated prevalence of 3.6% at 8 years of age (Wren et al., 2016). Prospective longitudinal studies have identified a number of predictors of PSD, including male sex, family history, atypical errors, lower socioeconomic status, hearing loss and suspected coordination difficulties (Eadie et al., 2015; Morgan et al., 2017; Wren et al., 2016) as well as associations between PSD and poorer literacy and educational outcomes, after accounting for biological sex, socioeconomic status and Intelligence Quotient (IQ) (Wren et al., 2021). It is not known whether there is an increased risk of SEBD for this group, or what the role of potential confounders such as biological sex, socioeconomic status and IQ might be. Identifying the relationships between PSD and SEBD can ultimately inform education and health services to ensure that children at risk are identified and offered appropriate support. The aim of the current study was therefore to address the current gap in the evidence to investigate the social, emotional and behavioural outcomes of children with PSD in older childhood using data from a large prospective, population‐based sample—the Avon Longitudinal Study of Parents and Children (ALSPAC)—including adjustment for biological sex, socio‐economic status, non‐verbal and verbal IQ. Three research questions drove the analyses:

- Are children with PSD at 8 years more likely to present with SEBD in older childhood than those without?
- To what extent do children with PSD at 8 years present with symptoms related to depression in older childhood?
- Are children with PSD at 8 years more likely to show antisocial and risk‐taking behaviours in older childhood than those without?

## METHODS

### Cohort study numbers

The Avon Longitudinal Study of Parents and Children (ALSPAC, www.bristol.ac.uk/alspac) is a transgenerational, observational. Population‐based study of health and development across the lifespan. Pregnant women resident in Avon, UK with expected dates of delivery between 1 April 1991 and 31 December 1992 were invited to take part. The initial number of pregnancies enroled was 14,541 and of these, there were a total of 14,676 foetuses, resulting in 14,062 live births and 13,988 children who were alive at 1 year of age.

Detailed information on parents and children in the ALSPAC sample is available from Fraser et al. (2013) and Boyd et al. (2013). Data have been collected via self‐report questionnaires, face‐to‐face clinical assessments, birth, medical, and educational records, and from biological samples, all collected prospectively at multiple timepoints during pregnancy and throughout childhood. From the age of 7, the entire cohort was invited to attend clinics for direct assessment of growth and development on an annual basis. Speech was assessed at the 8‐year clinic.

The study website contains details of all the data that are available through a fully searchable data dictionary and variable search tool at http://www.bristol.ac.uk/alspac/researchers/our‐data/.

### Participants

A total of 7390 children attended the clinic at 8 years of age, where a speech assessment was conducted, including a recording of continuous speech by trained assessors. Observation during the assessment identified 991 (13.4%) children as having some unusual features in their speech which could qualify them as atypical. A total of 580 (7.8%) of these demonstrated only errors that can be classified as common clinical distortions (Shriberg, 1993). The recordings of the remainder of those identified with atypical speech (*N* = 411, 5.5%) were transcribed and analysed by speech and language therapists to determine percentage consonants correct scores (Shriberg et al., 1997). This process was repeated for a random sample of 50 children from the rest of the cohort to provide normative data. The PSD group comprised those children whose percentage consonants correct scores, were more than 1.2 standard deviations below the mean (*N* = 263, 3.6%). Those whose speech was identified as atypical but whose percentage consonants correct score did not reach this threshold and those whose errors were limited to common clinical distortions were excluded from further analysis. This was because previous research on the dataset has revealed distinct differences in the profiles of children within these groups with regards to demographic, cognitive and speech motor skills, suggesting that they should not be considered equivalent to the rest of the cohort (Wren et al., 2012). Moreover, we were keen to explore the associations for those children with the most impacted speech production.

The total sample size for this study was therefore 6662 children, consisting of a case group of children with confirmed PSD (*N* = 263) and a control group comprising the rest of the cohort (*N* = 6399).

### Measures

Outcomes for behaviour and depression were measured using the Strengths and Difficulties Questionnaire (SDQ, Goodman, 1997) at age 10–11 and an adapted version of the Short Moods and Feelings Questionnaire (sMFQ, Angold et al., 1995) at age 10. Outcomes for antisocial and risk‐taking behaviours were measured using a range of interview questions during clinics at ages 10, 11 and 14.

The SDQ is a brief behavioural screening tool which can be completed by parents and teachers in 5 min for children aged 4–17. It comprises 25 questions and includes positive and negative attributes across five subscales: emotional symptoms, conduct problems, hyperactivity/inattention, peer relationship problems and prosocial behaviour. The SDQ has been validated against existing tools—the Child Behaviour Checklist (Achenbach, 1991) and the Rutter questionnaires (Elander & Rutter, 1996)—and shown to be able to distinguish between low‐ and high‐risk groups (Goodman, 1997; Goodman & Scott, 1999). It has also been shown to be better than the Rutter questionnaires at identifying strengths and pro‐social behaviours, and problems with inattention and peer relationships (Goodman, 1997). Follow‐up interviews with parents of children from the high‐risk groups suggested that the SDQ was better than the Child Behaviour Checklist at detecting problems with inattention and hyperactivity and as good at it as identifying internalising and externalising problems (Goodman & Scott, 1999).

The sMFQ is a self‐report screening measure of childhood depression, designed for use in epidemiological studies. It is a unifactorial scale comprising 13 items. It has been validated against the Children's Depression Inventory (Kovacs, 1983) and the Diagnostic Interview Schedule for Children depression scale (Costello et al., 1982), showing an ability to distinguish between children with depression and controls (Angold et al., 1995).

### Behaviour and depression

Parents completed the SDQ when their children were aged 11 and teachers were asked to complete it when the children were in their final year of primary education, aged 10–11. The SDQ scores were not normally distributed, so scores were dichotomised such that those in the bottom 10% of scores (highest 10% for prosocial) are considered problematic and the reference group is the top 90% (bottom 90% for prosocial).

At age 10, the sMFQ statements were read aloud to the child who was asked to indicate how they had been feeling or acting in the previous two weeks, and whether the statement was ‘true’, ‘sometimes true’ or ‘not at all true’. Scores of 2, 1 and 0 were allocated to each response respectively and summed together (range 0–26). As with the SDQ, scores were not normally distributed and so were dichotomised into children whose scores suggested that they were in a depressed category (score of 12 or above, Angold et al., 1995) and those not.

### Antisocial and risk‐taking behaviours

Outcomes relating to various antisocial behaviours were collected at age 10 via structured interviews where children were told they would be asked some questions about whether their friends or they had done something that could get them into trouble. They were assured of confidentiality and also told that everybody would be asked the same questions. They were first asked if their friends had taken part in a particular activity and then asked if they had taken part. A composite score for antisocial behaviour was derived from the responses to 11 questions asking about deliberately missing school, destroying something for fun, setting fire to something, stealing, getting into fights, cruelty to animals, being in trouble with the police, smoking cigarettes, drinking alcohol, being offered drugs and smoking cannabis. An overall antisocial score was derived (range).

At the age of 14, children completed a questionnaire where they were asked if they had ever smoked cigarettes.

Two of the antisocial and risk‐taking behaviours (tried alcohol at age 10; tried smoking at age 14) were binary yes/no responses where ‘no’ was the reference group, while the antisocial score at age 10 was dichotomised such that a score of 0 provided the reference group.

### Potential confounders

Biological sex, as recorded in birth records, and socio‐economic status, based on highest level of maternal education (specifically, whether mothers' education at finished at the end of compulsory schooling at age 16 or whether they had gone on to complete optional schooling to 18 or had completed degree level education), were included as confounders in all analyses. Analyses were also adjusted for performance on the Wechsler Intelligence Scale for Children (WISC III—Wechsler et al., 1992). Specifically, scores for each of the verbal and performance subtests as well as total IQ scores were used to ensure that neither general intelligence nor language skill could account for the results. The following sub‐tests were administered by trained psychologists: Information, Similarities, Arithmetic, Vocabulary, and Comprehension (verbal subtests), Picture Completion, Coding, Picture Arrangement, Block Design and Object Assembly (performance subtests). Children were assessed on the WISC on the same day as the speech samples were collected.

### Statistical analysis

For dichotomous outcomes, logistic regression was performed and odds ratios (ORs) and 95% confidence intervals (CIs) are presented. For each of the SDQ subscales we considered the ‘non‐problematic’ 90% of the population as the reference group. For continuous outcomes, linear regression was performed with regression coefficients and 95% CIs presented.

A number of adjusted models are presented (model 0 being unadjusted). Model 1 adjusted for biological sex and maternal education. In model 2 either performance IQ (model 2a) or verbal IQ (model 2b) was added. Finally model 3 added total IQ. At each point, all available data were used. All analyses were conducted using Stata (Version 13 Stata Corp, Texas, USA).

## RESULTS

The available sample size for each of the outcomes and confounders are summarised in Table 1. Compared with the baseline cohort (children alive at 1 year of age), those who attended the 8‐year clinic were more likely to be female and to have mothers with higher levels of education.

**TABLE 1:** Summary of variables and sample size

Variable name	Variable type	Variable source	Age when collected	Number available	
				Controls	Cases
Well‐being—Parent report					
Prosocial	Outcome	Strengths and Difficulties Questionnaire	11	5008	199
Hyperactivity	Outcome	Strengths and Difficulties Questionnaire	11	5000	197
Emotionality	Outcome	Strengths and Difficulties Questionnaire	11	5001	197
Conduct	Outcome	Strengths and Difficulties Questionnaire	11	5008	198
Peer problems	Outcome	Strengths and Difficulties Questionnaire	11	5008	199
Well‐being—Teacher report					
Prosocial	Outcome	Strengths and Difficulties Questionnaire	10–11	3677	144
Hyperactivity	Outcome	Strengths and Difficulties Questionnaire	10–11	3617	144
Emotionality	Outcome	Strengths and Difficulties Questionnaire	10–11	3617	144
Conduct	Outcome	Strengths and Difficulties Questionnaire	10–11	3616	144
Peer problems	Outcome	Strengths and Difficulties Questionnaire	10–11	3617	144

Depression	Variable type	Variable source	Age when collected	Number available	
				Controls	Cases
Depression	Outcome	Short Moods and Feelings Questionnaire	10	5483	209

Anti‐social/risk taking behaviours	Variable type	Variable source	Age when collected	Number available	
				Controls	Cases
Tried alcohol	Outcome	Item from anti‐social questionnaire	10	5492	209
Smoking	Outcome	Questionnaire item	14	3986	162
Anti‐social score	Outcome	Anti‐social questionnaire	10	5660	222

Confounders	Variable type	Variable source	Age when collected	Number available	
				Controls	Cases
Biological sex	Confounder	Midwifery records	Antenatal	6399	263
Highest level of maternal education reported by the mother	Confounder	Mother's questionnaire at 32 weeks gestation	Antenatal	5924	242
Performance IQ (subscale of WISC)	Confounder	Wechsler Intelligence Scale for Children	8 years clinic	6309	258
Verbal IQ (subscale of WISC)	Confounder	Wechsler Intelligence Scale for Children	8 years clinic	6319	257
Combined IQ (total of WISC)	Confounder	Wechsler Intelligence Scale for Children	8 years clinic	6292	256

Abbreviations: IQ, intelligence quotient; WISC, Wechsler Intelligence Scale for Children.

Descriptive statistics for the confounding variables are summarised in Table 2. There were significantly more boys in the case group as compared with controls (*p* < 0.001). There was a higher proportion of mothers with lower levels of education in the case group (*p* = 0.019). Cases had lower mean performance IQ score (*p* = 0.009), verbal IQ scores (*p* = 0.038) and total IQ scores (*p* = 0.003). Thus, all of these variables were controlled for in the analyses.

**TABLE 2:** Summary of descriptive statistics for the confounders

Confounder	Controls	Cases
Biological sex		
Male (49.0%)	3096 (48.4%)	169 (64.3%)
Female (51.0%) *p* < 0.001	3303 (51.6%)	94 (35.7%)
Maternal education		
<O level (21.7%)	1265 (21.4%)	70 (28.9%)
O level^a^ (35.2%)	2093 (35.3%)	79 (32.6%)
A levels^b^ or higher (38.4%) *p* = 0.019	2566 (43.3%)	93 (38.4%)
Performance IQ		
Mean (sd) *p* = 0.009	107.2 (16.6%)	101.0 (18.9%)
Verbal IQ		
Mean (sd) *p* = 0.038	99.8 (17.0%)	93.9 (19.0%)
Total IQ		
Mean (sd) *p* = 0.003	104.3 (16.3%)	97.6 (18.9%)

Abbreviation: IQ, intelligence quotient.

^a^ Compulsory examssinations taken at age 16.

^b^ Optional examinations taken at age 18.

Table 3 presents the results for behaviour and depression. The OR gives the increase or decrease in odds of being in the ‘problematic’ category (top 10% for each subscale of the SDQ, except prosocial [bottom 10%] or in the ‘depressed’ category for the mental health measure) for cases compared with controls.

**TABLE 3:** Wellbeing and Mental Health Outcomes: odds ratios (ORs) (95% confidence intervals [CIs]) for being in the problematic category for each sub‐scale of Strengths and Difficulties Questionnaire (SDQ) and for being depressed as defined by the Short Moods and Feelings Questionnaire (sMFQ) for cases versus controls

	Model 0: Unadjusted		Model 1: Adjusted for biological sex and maternal education		Model 2a: Adjusted for biological sex, maternal education and performance intelligence quotient (IQ)		Model 2b: Adjusted for biological sex, maternal education and verbal IQ		Model 3: Adjusted for biological sex, maternal education, verbal and performance IQ	
Outcome	OR [95% CI]	*p*‐value	OR [95% CI]	*p*‐value	OR [95% CI]	*p*‐value	OR [95% CI]	*p*‐value	OR [95% CI]	*p*‐value
SDQ: Parent report (age 11)										
Prosocial [*n* = 805/5257]	1.58 [1.32, 2.23]	0.009	1.40 [0.98, 2.01]	0.066	1.33 [0.92, 1.93]	0.125	1.36 [0.94, 1.96]	0.106	1.36 [0.94, 1.96]	0.105
Hyperactivity [*n* = 360/5197]	1.28 [0.77, 2.13]	0.339	1.12 [0.66, 1.50]	0.683	0.99 [0.57, 1.71]	0.958	0.89 [0.51, 1.57]	0.692	0.89 [0.50, 1.57]	0.688
Emotionality [*n* = 341/5195]	1.27 [0.75, 2.15]	0.368	1.25 [0.72, 2.20]	0.43	1.12 [0.63, 2.01]	0.703	1.15 [0.64, 2.05]	0.873	1.14 [0.63, 2.04]	0.668
Conduct [*n* = 372/5206]	0.99 [0.57, 1.72]	0.967	0.81 [0.45, 1.48]	0.498	0.78 [0.43, 1.42]	0.413	0.77 [0.42, 1.41]	0.399	0.76 [0.42, 1.39]	0.377
Peer problems [*n* = 427/5207]	2.42 [1.65, 3.55]	<0.001	2.07 [1.37, 3.11]	<0.0001	1.90 [1.25, 2.90]	0.003	1.81 [1.18, 2.78]	0.007	1.81 [1.18, 2.78]	0.007
SDQ: Teacher report (age 10–11)										
Prosocial [*n* = 337/3761]	1.78 [1.11, 2.87]	0.017	1.53 [0.93, 2.50]	0.094	1.44 [0.87, 2.40]	0.157	1.36 [0.8, 2.27]	0.243	1.36 [0.81, 2.27]	0.243
Hyperactivity [*n* = 389/3761]	1.78 [1.14, 2.80]	0.012	1.45 [0.90, 2.35]	0.125	1.22 [0.74, 2.03]	0.435	1.12 [0.68, 1.85]	0.652	1.08 [0.56, 1.80]	0.754
Emotionality [*n* = 414/3761]	1.93 [1.25, 2.97]	0.003	2.05 [1.31, 3.20]	0.002	1.94 [1.24, 306]	0.004	1.83 [1.16, 2.89]	0.01	1.80 [1.14, 2.85]	0.012
Conduct [*n* = 329/3760]	1.52 [0.91, 2.52]	0.107	1.18 [0.68, 2.03]	0.561	1.12 [0.64, 1.95]	0.696	1.07 [0.62, 1.87]	0.805	1.05 [0.60, 1.83]	0.954
Peer problems [*n* = 383/3761]	2.78 [1.85, 4.16]	<0.0001	2.60 [1.71, 3.96]	<0.0001	2.37 [1.54, 3.66]	<0.0001	2.38 [1.54, 3.68]	<0.0001	2.30 [1.49, 3.57]	<0.0001
Short Moods and Feelings Questionnaire: Child report (age 10)										
Depression score: Child report [*n* = 568/5692]	1.73 [1.18, 2.54]	0.005	1.39 [0.91, 2.14]	0.127	1.20 [0.77, 1.87]	0.434	1.14 [0.72, 1.80]	0.583	1.10 [0.70, 1.75]	0.653

*Note*: *N* varies according to outcome: model 0 = 3498–5782; model 1 = 3450–5309; model 2a = 3452–5317; model 2b = 3452–5326; model 3 = 3440–5302.

The unadjusted models for the SDQ showed that case children were more likely to be rated by parents as being in the problematic group for prosocial (unadjusted OR: 1.58 [95% CI: 1.32–2.23]) and peer problems (unadjusted OR: 2.42 [95% CI: 1.65–3.55]). After taking confounders into account, the association with prosocial disappeared; however, the association with peer problems remained albeit slightly attenuated (e.g., model 3: adjusted OR 1.81 [95% CI: 1.18–2.78]).

The unadjusted models for the SDQ showed that case children were more likely to be rated by teachers as being in the problematic group for more subscales: prosocial (unadjusted OR: 1.78 [95% CI: 1.11–2.87]); hyperactivity (unadjusted OR: 1.78 [95% CI: 1.14–2.80]); emotionality (unadjusted OR: 1.93 [95% CI: 1.25–2.97]); and peer problems (unadjusted OR: 2.78 [95% CI: 1.85–4.16]). The associations for teacher report of prosocial behaviour and hyperactivity were no longer evident in any of the adjusted models. However, strong associations remained, particularly for peer problems, with the fully adjusted OR attenuated to 2.30 [95% CI: 1.49–3.57]. The association with emotionality was also attenuated but remained after full adjustment (OR = 1.80 [956% CI: 1.14, 2.85]).

The unadjusted model (model 0) for depression showed that case children were more likely to be classified as depressed compared with controls (OR: 1.73 [95% CI: 1.18–2.54]), but the association was no longer evident in any of the adjusted models.

Table 4 presents the results of the associations with antisocial and risk‐taking behaviours. No associations were evident.

**TABLE 4:** Anti‐social and risk taking behaviours: odds ratios (ORs) (95% confidence intervals [CIs]) for being in the reference category for cases versus controls

	Model 0: Unadjusted		Model 1: Adjusted for biological sex and maternal education		Model 2a: Adjusted for biological sex, maternal education and performance intelligence quotient (IQ)		Model 2b: Adjusted for biological sex, maternal education and verbal IQ		Model 3: Adjusted for biological sex, maternal education, verbal and performance IQ	
Outcome	OR [95% CI]	*p*‐value	OR [95% CI]	*p*‐value	OR [95% CI]	*p*‐value	OR [95% CI]	*p*‐value	OR [95% CI]	*p*‐value
Tried alcohol age 11 (no) [*n* = 5602/5701]	0.82 [0.26, 0.61]	0.735	0.46 [0.11, 1.88]	0.279	0.44 [0.11, 1.79]	0.435	0.44 [0.11, 0.81]	0.254	0.43 [0.10, 0.76]	0.238
Antisocial score age 10 (0) [*n* = 4955/5782]	1.01 [0.69, 1.48]	0.961	0.71 [0.47, 1.08]	0.105	5.70 [0.46, 1.06]	0.092	0.71 [0.47, 1.07]	0.103	0.70 [0.46, 1.06]	0.090
Smoking age 14 (no) [*n* = 3102/4148]	0.78 [0.53, 1.15]	0.207	0.73 [0.48, 1.11]	0.143	0.70 [0.45, 1.06]	0.093	1.73 [0.48, 1.10]	0.133	0.69 [0.45, 1.06]	0.093

*Note*: *N*: model 0 = 2325–5782; model 1 = 2188–5387; model 2a = 2164–5317; model 2b = 2170–5326; model 3 = 2159–5302.

## DISCUSSION

This study investigated whether PSD was associated with SEBD using data from a large, prospective, population‐based sample. Following adjustment for biological sex, socio‐economic status and verbal and performance IQ, children with PSD at age 8 were more likely to show peer problems at age 10–11 as reported by teachers and parents. Teachers were also more likely to report problems with emotionality. Children with PSD were found to be more likely to report symptoms of depression than their peers though this was explained by the confounders of biological sex, maternal education, performance and verbal IQ rather than their speech status. They were no more likely to become involved in antisocial and risk‐taking behaviour in later childhood and early adolescence than their peers.

### Are children with PSD more likely to present with SEBD?

Children with PSD at age 8 were more likely to present with certain types of SEBD but not all. They were more likely to present with peer problems at age 10–11 as reported by teachers and parents even after controlling for biological sex, maternal education, verbal and performance IQ. Teacher's reports of emotionality and peer problems was more likely to be associated with case status after adjustment, suggesting children might show different behaviours in school or that certain behaviours may be perceived differently at home and at school.

These findings are consistent with previous studies that have identified an association between the broader group of children with SLCN and difficulties with peer relationships and bullying (Durkin et al., 2017; Forrest et al., 2018; McCormack et al., 2009, 2011) and emotionality and hyperactivity (Conti‐Ramsden et al., 2013; Lindsay & Dockrell, 2000).

Findings from the Millennium Cohort Study have suggested that peer problems may be a mediator between language disorder and emotional difficulties such that positive peer relationships act as a protective factor (Forrest et al., 2018), while meta‐analyses of both cross‐sectional and longitudinal studies have demonstrated significant relationships between peer victimisation and internalising problems (Hawker & Boulton, 2000; Reijntjes et al., 2010). In this study however, there was stronger evidence of an association with peer problems, some evidence for an association with emotionality in school and no evidence of an association with behavioural problems. This profile was unexpected and is in contrast with broader findings of increased rates of behavioural problems in other profiles of SLCN, such as children with DLD (Conti‐Ramsden et al., 2013; St Clair et al., 2011). Our findings may suggest a different and more specific link between communication difficulties as a result of PSD and peer problems, although it is also possible that emotional and behavioural difficulties may become more apparent at a later stage in adolescence, not assessed in the current study. For example, Forrest et al. (2018) found that emotional difficulties that were identified in adolescence could partly be accounted for by peer problems earlier in childhood in DLD. This large‐scale population study, with a carefully defined group of children with PSD that included adjustment for language provides new insights into the specific relationships that may exist between PSD and later psychosocial outcomes. Previous studies that have compared children with speech sound disorder alone with children with speech sound disorder with comorbid language impairment or language impairment alone have found that associations with later SEBD and psychiatric disorders were related to difficulties with language rather than speech (Beitchman et al., 2001; Lewis et al., 2016).

### To what extent do children with PSD present with symptoms related to depression?

When all confounders were taken into account, children with PSD were no more likely to report symptoms of depression than their peers at age 10. While this is encouraging, depression typically presents in mid‐adolescence (Orchard et al., 2017), so it is possible that there was not enough variability in symptoms at age 10, and that this relationship may emerge later in childhood. Furthermore, there is some indication in the literature that parents and children report different symptom severity (Eg et al., 2018; Orchard et al., 2019), with a lack of clarity regarding which report is more ‘accurate’. Future work would benefit from examining parental report as well as child report (De Los Reyes et al., 2013) and considering children's reports in older adolescence.

### Are children with PSD more likely to show antisocial and risk‐taking behaviours?

No associations were observed between PSD and risk of antisocial behaviour, trying alcohol at age 10, or smoking cigarettes at age 14. This is similar to Beitchman et al. (2001) who found no evidence of increased rates of substance abuse and antisocial personality disorder in adolescents with a history of speech problems in early childhood. As with the measures for depression however, it is possible that associations might be seen at slightly older ages, particularly for trying alcohol.

### Limitations of this study

Whilst there were some clear strengths of this study such as the large sample, prospective design and diverse range of SEBD measures, it is important to note some key limitations. Firstly, direct assessment was used to determine case status for this study but nevertheless, diagnosis of PSD was not confirmed by clinical assessment. Moreover, we did not exclude children with PSD and other comorbidities which may have confounded our results. Secondly, although the sample size used in the analysis was substantial in comparison to other epidemiological studies (even after missing data), it lacked the diversity seen in the UK population today. The results would be strengthened by replicating with a more diverse sample. Thirdly, the findings presented could be explained by residual confounding. While we have adjusted for known factors, there may be other unknown factors which have impacted on the associations observed but which we have not yet identified. Fourthly, the measures used for behaviour and depression outcomes, the SDQ and the sMFQ are screening assessments rather than clinical diagnostic tools. Nevertheless they were designed specifically for the purposes of large‐scale epidemiological research where clinical assessment is not feasible. Finally, the analyses reported a number of outcome measures from a range of different timepoints but none of the measures were themselves repeated, thus providing a series of cross‐sectional analyses using a longitudinal dataset. Future work could consider some of the questions arising from this work such as whether signs of depression or early use of alcohol are observed in children with PSD when they are older and also whether the problems observed in this cohort with peer relationships become more noticeable as children progress through school.

### Clinical implications

This study highlights the importance of identifying children with PSD, including those with a history of PSD, throughout their primary and secondary school years in order to ensure appropriate monitoring and support are in place. Speech and Language Therapists, education and health staff need to be aware that children with PSD are at risk of experiencing difficulties with peer relationships and emotionality in school, in addition to poorer education outcomes (Wren et al., 2021), and that the profile of these needs may change over time. Our findings support intervention for PSD in early childhood due to the potential that improvement in speech may lead to a reduction in the negative sequelae seen in older childhood and adolescence. This study has also raised important implications for future research in investigating SEBD in older children with PSD to examine later emerging wellbeing and mental health difficulties, which have been reported in other populations of children with SLCN (Beitchman et al., 2001, 2014; Botting et al., 2016; Conti‐Ramsden & Botting, 2008; Wadman et al., 2011). This future research is also important as emotional problems in childhood have been associated with increased risk of a range of negative health and wellbeing outcomes in later life (Bridge et al., 2006; Clayborne et al., 2019; Essau et al., 2014; Keenan‐Miller et al., 2007; Woodward et al., 2001).

## CONCLUSIONS

Children with PSD at 8 years of age show an increase in SEBD between the ages of 10 and 14 in specific areas, particularly problems with peer relationships and emotionality in school, compared with peers according to their parents and teachers. However, they appear to be no more likely than their peers to report depressive symptoms at age 10 or to become involved in antisocial and risk‐taking behaviour at age 11–14. Whilst it is important to be aware that the findings may be affected by the respondent (for example, parent, child or teacher) or the age of measurement, the results do suggest that PSD can lead to some problems with SEBD. Professionals working with children with PSD should be aware of the potential for difficulties in peer relationships and be mindful of the possibility of an impact on wellbeing in older childhood as well as on educational outcomes.

## AUTHOR CONTRIBUTIONS

**Yvonne Wren**: Investigation; Project administration; Writing – original draft. **Emma Pagnamenta**: Writing – original draft; Writing – review & editing. **Faith Orchard**: Writing – original draft; Writing – review & editing. **Tim J. Peters**: Supervision; Writing – review & editing. **Alan Emond**: Conceptualization; Funding acquisition. **Kate Northstone**: Formal analysis; Writing – review & editing. **Laura Louise Miller**: Formal analysis; Writing – review & editing. **Susan Roulstone**: Conceptualization; Funding acquisition; Methodology; Project administration; Supervision; Writing – review & editing.

## CONFLICT OF INTEREST

The authors have declared that they have no competing or potential conflicts of interest.

## ETHICAL CONSIDERATIONS

Ethical approval for the study was obtained from the ALSPAC Ethics and Law Committee and the Local Research Ethics Committees http://www.bristol.ac.uk/alspac/researchers/research‐ethics/. Informed consent for the use of data collected via questionnaires and clinics was obtained from participants following the recommendations of the ALSPAC Ethics and Law Committee at the time.

## Data Availability

The datasets generated during and/or analysed during the current study are not publicly available but access can be requested via a proposal to the ALSPAC study team.
